# Pretreatment with valproic acid alleviates pulmonary fibrosis through epithelial–mesenchymal transition inhibition in vitro and in vivo

**DOI:** 10.1038/s41374-021-00617-2

**Published:** 2021-06-24

**Authors:** Lin Chen, Azeem Alam, Aurelie Pac-Soo, Qian Chen, You Shang, Hailin Zhao, Shanglong Yao, Daqing Ma

**Affiliations:** 1 Department of Anesthesiology, Institute of Anesthesiology and Critical Care Medicine, Union Hospital, Tongji Medical College, Huazhong University of Science and Technology, Wuhan, China; 2 Anesthetics, Pain Medicine and Intensive Care, Department of Surgery and Cancer, Faculty of Medicine, Imperial College London, Chelsea & Westminster Hospital, London, UK; 3 Department of Critical Care Medicine, Institute of Anesthesiology and Critical Care Medicine, Union Hospital, Tongji Medical College, Huazhong University of Science and Technology, Wuhan, China

**Keywords:** Cell division, Immunochemistry

## Abstract

Epithelial–mesenchymal transition (EMT) plays a crucial role in the development of pulmonary fibrosis. This study aims to investigate the effects of valproic acid (VPA) on EMT in vitro and in vivo. In vitro, EMT was induced by the administration of transforming growth factor-β1 (TGF-β1) in a human alveolar epithelial cell line (A549). The dose effects of VPA (0.1–3 mM) on EMT were subsequently evaluated at different timepoints. VPA (1 mM) was applied prior to the administration of TGF-β1 and the expression of E-cadherin, vimentin, p-Smad2/3 and p-Akt was assessed. In addition, the effects of a TGF-β type I receptor inhibitor (A8301) and PI3K-Akt inhibitor (LY294002) on EMT were evaluated. In vivo, the effects of VPA on bleomycin-induced lung fibrosis were evaluated by assessing variables such as survival rate, body weight and histopathological changes, whilst the expression of E-cadherin and vimentin in lung tissue was also evaluated. A8301 and LY294002 were used to ascertain the cellular signaling pathways involved in this model. The administration of VPA prior to TGF-β1 in A549 cells prevented EMT in both a time- and concentration-dependent manner. Pretreatment with VPA downregulated the expression of both p-Smad2/3 and p-Akt. A8301 administration increased the expression of E-cadherin and reduced the expression of vimentin. LY294002 inhibited Akt phosphorylation induced by TGF-β1 but failed to prevent EMT. Pretreatment with VPA both increased the survival rate and prevented the loss of body weight in mice with pulmonary fibrosis. Interestingly, both VPA and A8301 prevented EMT and facilitated an improvement in lung structure. Overall, pretreatment with VPA attenuated the development of pulmonary fibrosis by inhibiting EMT in mice, which was associated with Smad2/3 deactivation but without Akt cellular signal involvement.

## Abstract (web summary)

This study investigated the effect of valproic acid (VPA) on epithelial–mesenchymal transition (EMT). In vitro, VPA prevents EMT in a time- and concentration-dependent manner. In vivo, pretreatment with VPA attenuates pulmonary fibrosis development through EMT inhibition in mice, which was associated with Smad2/3 deactivation but without Akt signal involvement.

## Introduction

Chemotherapy can trigger the development of lung fibrosis in a subset of patients [1, 2]. This type of lung fibrosis is characterized by various pathological hallmarks, including chronic pulmonary epithelial injury and the proliferation and activation of fibroblasts, which subsequently promote myofibroblast formation and extracellular matrix (ECM) accumulation [3]. For example, toxic chemical compounds such as asbestos and bleomycin can cause the death of lung epithelial cells and stimulate the production of reactive oxygen species and inflammatory mediators. As a result, repair mechanisms within the lung are activated which attempt to restore lung function. However, dysregulation of these processes may result in the initiation of fibrogenesis [4]. Recent studies have demonstrated that fibroblasts originating from bone marrow [5], endothelial [6], and epithelial cells [7] can all be activated to acquire the myofibroblast phenotype, thus resulting in the development of fibrosis.

Although the mechanisms underlying the development of non-idiopathic lung fibrosis remain to be elucidated, epithelial–mesenchymal transition (EMT) has been suggested as one of the key mechanisms involved in lung fibrosis, in general [8, 9]. EMT is defined as the process by which epithelial cells differentiate into fibroblast/myofibroblast-like cells [7, 8]. This process involves a decrease in various epithelial cell biomarkers such as E-cadherin and occludin, as well as a concomitant increase in mesenchymal cell biomarkers, such as vimentin and fibronectin [10]. EMT also results in a change in the polarity of epithelial cells, which subsequently acquire a spindle-like shape. EMT is thought to be closely linked to the development of tissue fibrosis in various organs, including the heart, kidney, lung and liver [11–14]. Transforming growth factor-β1 (TGF-β1), which is present at the site of fibrosis, plays an important role in inducing EMT [15]. By binding to its receptor, TGF-β1 classically activates Smad2/3 in the cytoplasm leading to the formation of p-Smad2/3. The latter binds to Smad4 and translocates to the nucleus, resulting in changes to gene transcription. In addition, noncanonical pathways including Notch and Wnt/β-catenin are also responsible for mediating EMT and the subsequent development of fibrosis [15, 16].

Valproic acid (VPA) has been used clinically as a broad-spectrum antiepileptic for seizures and as a mood stabilizer for bipolar disorder. Interestingly, VPA has been shown to demonstrate anti-inflammatory and antioxidant properties, as well as the ability to induce cancer cell apoptosis [17–19]. A clinical study demonstrated that the long-term use of VPA was associated with a reduced incidence of head and neck cancer in the American veteran population [20]. In animal studies, the administration of VPA has been shown to protect mice against sepsis-induced multiple organ dysfunction syndrome [21, 22], as well as ischemia–reperfusion-associated acute lung injury [23]. VPA has also demonstrated the ability to inhibit the progression of fibrosis in the liver [24] and kidney [25], as well as reduce the development of cardiac hypertrophy [26]. In a rat lung fibrosis model, VPA and butyrate, either on its own or in combination, inhibited cytokine release and oxidative stress, and ameliorated fibrosis caused by ECM deposition of fibroblasts [27]. However, the protective effects of VPA against EMT development associated with chemotherapy, as well as its underlying mechanisms, remain to be elucidated. In this study, we hypothesize that pretreatment with VPA mitigates bleomycin-induced lung fibrosis through EMT inhibition, which was investigated in both in vitro and in vivo settings.

## Materials and methods

### Cell culture

The alveolar epithelial cell line (A549), purchased from ATCC (Manassas, VA, USA), was cultured in RPMI-1640 culture medium supplemented with 10% fetal bovine serum, 100 U/ml penicillin, and 100 μg/ml streptomycin in a humidified atmosphere of 5% CO_2_ at 37 °C. A549 cells have been widely used in in vitro models of lung epithelial cell injury and EMT progression [28, 29].

### Cell treatment

EMT in A549 cells was induced by TGF-β1 (10 ng/ml, PeproTech, USA). The duration of A549 cell exposure to TGF-β1, as well as the dose of TGF-β1, is based on previous studies [28, 30]. Other culture cohorts were treated with VPA (Sigma, Munich, Germany) with or without 10 ng/ml TGF-β1 for 48 h as indicated. The following inhibitors were used wherever appropriate: PI3k-Akt inhibitor LY294002 (Cell signaling technology, Danvers, MA, USA) and TGF-β type I receptor inhibitor A8301 (Tocris, Oxford, UK). All cells were then fixed with paraformaldehyde for immunofluorescent staining or were used to extract proteins for western blot analysis.

### MTT assay

Cells were cultured in 96-well plates and, once they became 90% confluent, were treated with VPA with or without TGF-β1 for 48 h. The MTT assay was used to assess cell viability which required the addition of tetrazolium dye MTT 3-(4,5-dimethylthiazol-2-yl)-2,5-diphenyltetrazolium bromide to the culture medium and incubation for a further 4 h at 37 °C. The medium was then carefully removed and 100 μl of dimethyl sulfoxide was added to each well and mixed for 10 min at room temperature. The absorbance at 570 nm was determined using Multiskan FC (Thermo Fisher Scientific, Shanghai, China).

### Animal model

Male C57BL/6J mice, 8–10 weeks old weighing 22–25 g, were purchased from Hua Fu Kang Co. (Beijing, China). The mice were given standard laboratory chow and water *ad libitum* and housed in a pathogen-free room at a temperature between 22 and 24 °C with a 12 h light/dark cycle. All animal experimentations were approved by the Ethics Committee of Tongji Medical College of Huazhong University of Science and Technology. All studies have been reported in accordance with the ARRIVE guidelines for reporting experiments involving animals [31].

As previously reported by Kabel et al. [27] mice randomly received VPA (100 mg/kg) (*n* = 10) once daily for a week via intraperitoneal injection or LY294002 (5 mg/kg) or A8301 (1 mg/kg) (*n* = 10) once via intraperitoneal injection. The mice were then anaesthetized with an injection of 2% sodium pentobarbital intraperitoneally (80 mg/kg, Sigma, Munich, Germany). Endotracheal intubation was performed and lung fibrosis was induced by instilling bleomycin (2 mg/kg; once) *via* the endotracheal tube. Mice that received VPA prior to bleomycin administration were given VPA by intraperitoneal injection once daily for up to 28 days. The groups treated with LY294002 or A8301, as well the naïve control group, all received the same volume of normal saline for up to 28 days. The survival and body weight of the mice were closely monitored daily. Mice were subsequently sacrificed with a terminal overdose of sodium pentobarbital, and their lung tissue was collected for further analysis.

### Histopathological analysis of lung slices

Lung specimens from the mice were inflated to 15 cm H_2_O with 4% paraformaldehyde and subsequently embedded in paraffin. Lung slices were prepared and stained with hematoxylin and eosin. Masson staining was also performed to determine the development of collagen as previously reported [32]. The Ashcroft score [33] was then assessed by a researcher who was blinded to the experimental protocols.

### Western blot analysis

Total protein was either extracted from cultured cells 48 h after TGF-β1 administration, or from murine lung tissue. The protein concentration was determined using a BCA Protein Assay Kit (Thermo Fisher Scientific, Shanghai, China). Following this, 30 μg of protein/sample was separated by electrophoresis on 10% polyacrylamide sodium dodecyl sulfate gels and transferred to polyvinylidene difluoride membranes. The membranes were blocked for 1 h at room temperature with 5% nonfat milk and incubated overnight at 4 °C with the following antibodies: E-cadherin (1:1000, Santa Cruz, Dallas, TX, USA), vimentin (1:500, Santa Cruz, Dallas, TX, USA), p-Smad2/Smad3 (1:1000, Cell Signaling Technology, Danvers, MA, USA), Smad2/3 (1:1000, Cell Signaling Technology, Danvers, MA, USA), p-Akt (1:1000, Cell Signaling Technology, Danvers, MA, USA), Akt (1:1000, Cell Signaling Technology, Danvers, MA, USA), and GAPDH (1:1000, Millipore, Danvers, MA, USA). The next day, the membranes were washed three times in Tris-buffered saline with Tween 20 and incubated with goat-anti-rabbit antibody (1:2000, Cell Signaling Technology, Danvers, MA, USA) or goat-anti-mouse antibody (1:2000, Cell Signaling Technology, Danvers, MA, USA) for 1 h at room temperature. After washing, the membranes were incubated with chemiluminescence reagents (Santa Cruz, Dallas, TX, USA) and detected using a UVP imaging system (Upland, CA, USA). The images obtained were further analyzed by using Image J software (version 1.48 v; National Institutes of Health, USA).

### Immunofluorescent staining and analysis

For the in vitro component, the cells were washed with cold PBS three times and fixed in 4% paraformaldehyde at room temperature for 30 min. The cells were then blocked with 3% bovine serum albumin for 1 h at room temperature. For E-cadherin and vimentin staining, the cells were incubated overnight at 4 °C with rabbit anti E-cadherin antibody and rabbit anti-vimentin antibody (1:200, Santa Cruz, Dallas, TX, USA). On the next day, the cells were washed three times with cold PBS and incubated with Alexa Fluor 488-conjugated goat-anti-rabbit IgG (1:200, Jackson ImmunoResearch, West Grove, PA, USA) at 37 °C for 1 h. Subsequently, the cells were washed three times with cold PBS and incubated with 4′,6-diamidino-2-phenylindole (DAPI) for nuclear staining at room temperature for 10 min. Images were visualized using an Olympus IX71 fluorescence microscope (Olympus, Tokyo, Japan). For the in vivo component, the paraffin-embedded lung sections were deparaffinized and rehydrated before staining. The mean intensity of fluorescence obtained from the different groups was analyzed using Image J software.

### Statistical analysis

Data are expressed as dot plot and mean ± standard error of the mean (SEM) and were analyzed using one-way variance analysis, followed by the post hoc Tukey test. Survival was analyzed using the Kaplan–Meier test. All the statistical analyses were performed using GraphPad Prism 5 (GraphPad Software, San Diego, CA, USA). A *P* value < 0.05 was considered to be statistically significant.

## Results

### VPA-mediated inhibition of TGF-β1-induced EMT in alveolar epithelial cells is time- and concentration-dependent

**Fig. 1:**
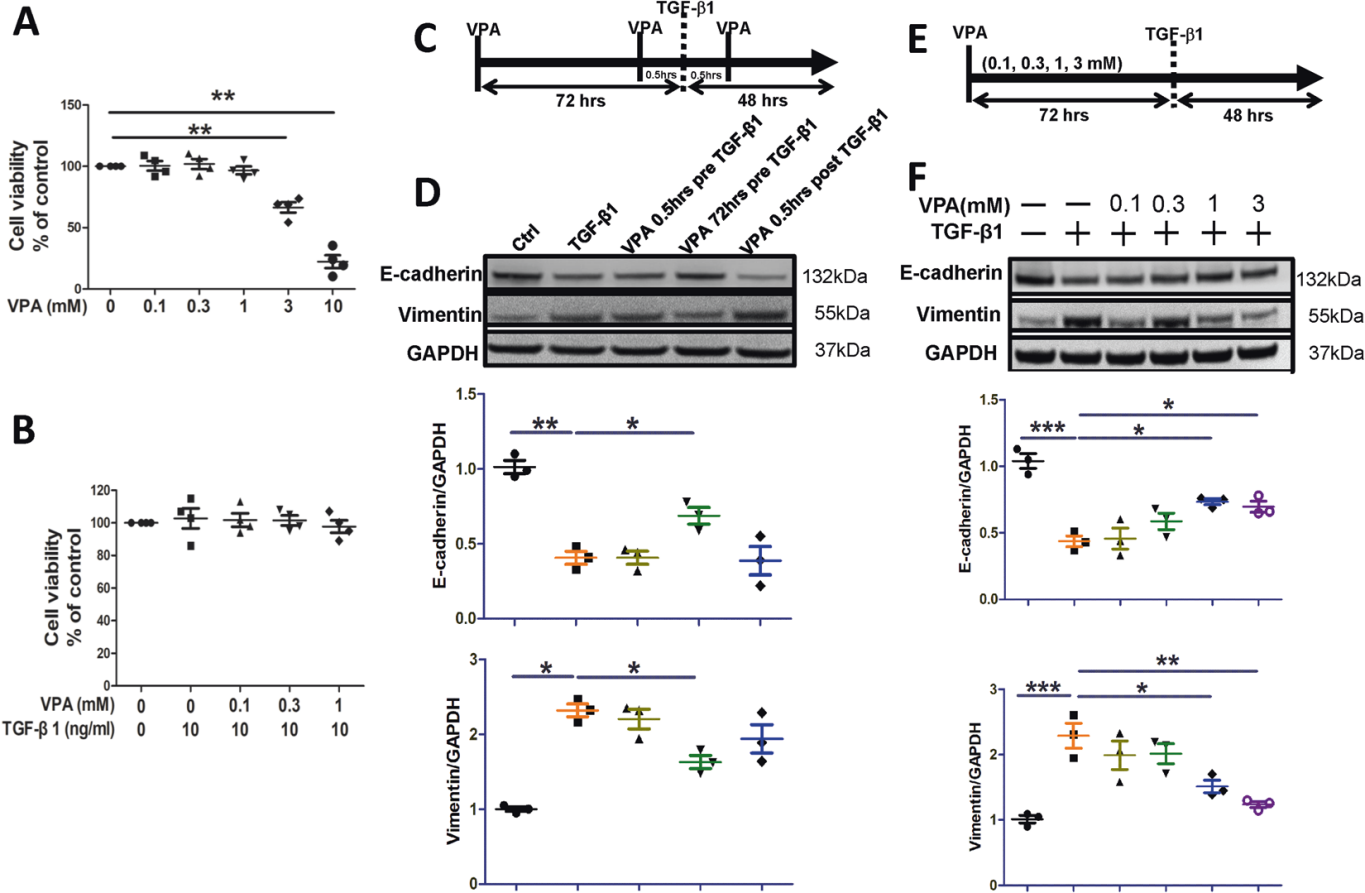
VPA inhibits EMT in alveolar epithelial cells (A549) in a time- and concentration-dependent manner. **A** A549 cells were treated with VPA (0–10 mM) for 48 h, and cell viability was assessed using an MTT assay. **B** A549 cells were treated with different concentrations of VPA with or without TGF-β1 (10 ng/ml) for 48 h and cell viability was assessed using an MTT assay. **C** The time-dependent effect of VPA: 1 mM VPA was administered 0.5 or 72 h prior to TGF-β1 and 0.5 h post TGF-β1, and cells were harvested 48 h after TGF-β1 administration and used for further analysis. **D** The expression of E-cadherin and vimentin and GAPDH serves as the loading control. **E** The concentration-dependent effect of VPA: VPA (0.1, 0.3, and 1 mM) was administered 72 h prior to TGF-β1 and cells were harvested 48 h after TGF-β1 administration and used for further analysis. **F** The expression of E-cadherin and vimentin in various conditions. Data are presented as dot plot and mean ± SEM (*n* = 3). *P < 0.05; **P < 0.01; ***P < 0.001.

Firstly, we determined the effects of different concentrations of VPA on the viability of alveolar epithelial cells. VPA did not cause alveolar epithelial cell death at concentrations of 0.1, 0.3, or 1 mM. However, 3 mM VPA and 10 mM VPA reduced alveolar epithelial cell viability to 67% (*p* < 0.01) and 23% (*p* < 0.01) of that of the control group, respectively (Fig. 1A). In addition, A549 cells incubated with VPA (0.1, 0.3, and 1 mM) and TGF-β1 at 10 ng/ml demonstrated no significant change in cell viability (Fig. 1B). We also applied VPA at various timepoints to determine whether VPA inhibited EMT in a time-dependent manner. 1 mM VPA was administered for 0.5 h or 72 h prior to TGF-β1, and 0.5 h post-TGF-β1 (Fig. 1C). Compared to the TGF-β1 group, pretreatment with VPA for 72 h inhibited EMT, as demonstrated by a significant increase in the expression of E-cadherin (*p* < 0.05), alongside a concurrent downregulation in the expression of vimentin (*p* < 0.05) (Fig. 1D). Furthermore, we attempted to identify the effective concentration of VPA in preventing EMT. Different concentrations of VPA at 0.1, 0.3, 1, or 3 mM were administered for 72 h prior to TGF-β1 (Fig. 1E). Both 1 mM and 3 mM VPA were able to increase E-cadherin expression (*p* < 0.05) and reduce vimentin expression (*p* < 0.05) compared to the TGF-β1 cohort (Fig. 1F). However, VPA was not effective at concentrations <1 mM (Fig. 1F).

**Fig. 2:**
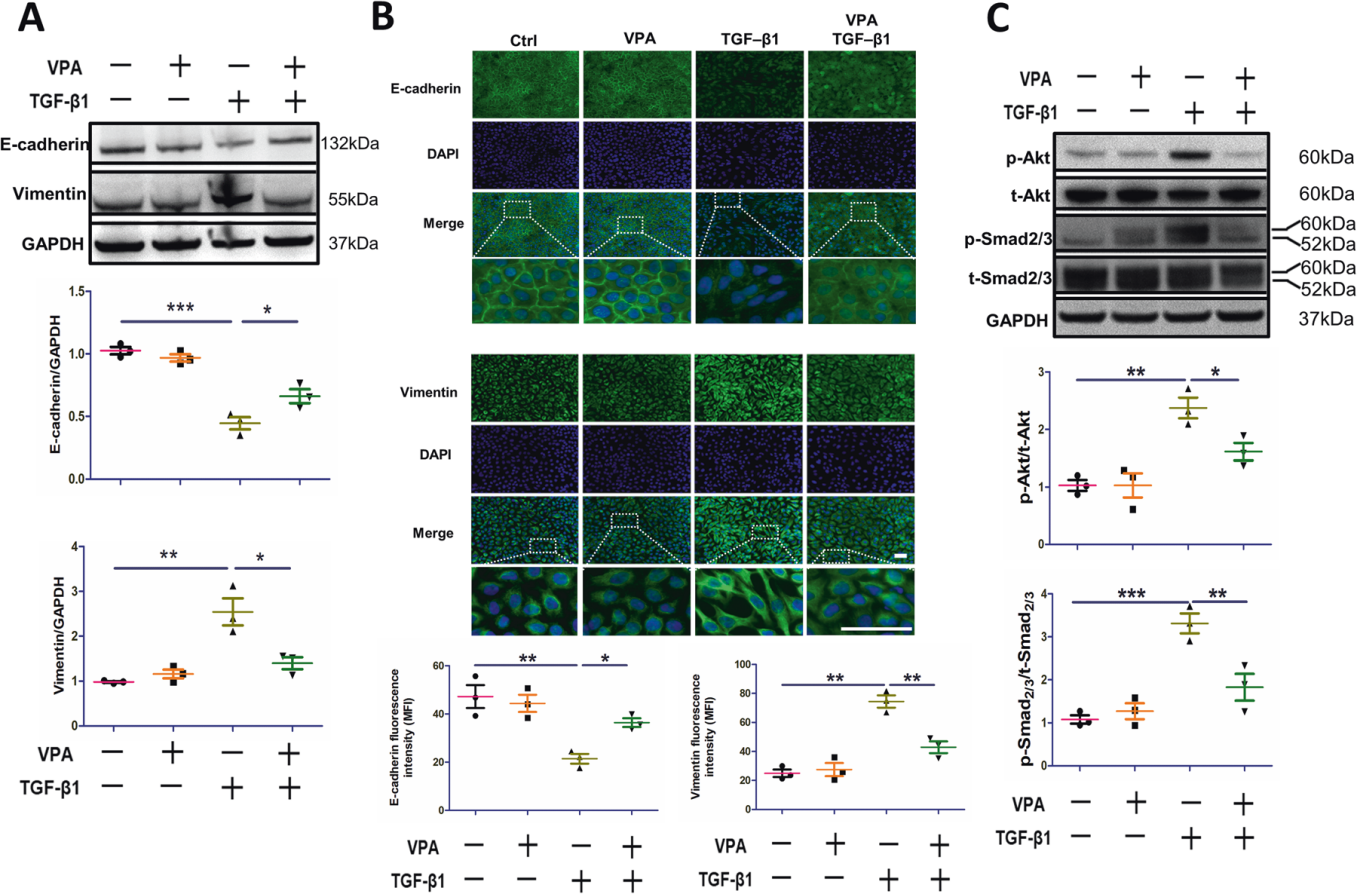
Pretreatment with VPA inhibits EMT in alveolar epithelial cells. A549 cells were treated with VPA (1 mM) 72  h prior to TGF-β1, and cells were collected 48  h after TGF-β1 stimulation. **A** The expression of E-cadherin and vimentin in various conditions, GAPDH serves as the loading control. **B** The expression of E-cadherin and vimentin in various conditions was analyzed by immunofluorescent staining. Mean fluorescence intensity was determined by Image J software. **C** The expression of p-Akt and p-Smad2/3 in various conditions. Data are presented as dot plot and mean ± SEM (*n* = 3). Scale bar = 50 μm. *P < 0.05; **P < 0.01; ***P < 0.001.

Having identified the time and concentration of VPA required to prevent EMT, the effects of VPA during EMT were subsequently validated and an assessment of its associated signaling pathways was performed. Western blot and immunofluorescent staining were used to analyze the expression of E-cadherin and vimentin. TGF-β1-induced EMT in A549 cells compared to the control group (Fig. 2A). In comparison to the TGF-β1 cohort, pretreatment with 1 mM for 72 h significantly increased the expression of E-cadherin (*p* < 0.05) and downregulated vimentin expression (*p* < 0.05). Immunofluorescence staining demonstrated similar results (Fig. 2B). Surprisingly, during EMT induced by TGF-β1, the expression of p-Akt and p-Smad2/3 increased compared to the control group, whilst pretreatment with VPA downregulated both p-Akt (*p* < 0.05) and p-Smad2/3 (*p* < 0.01) (Fig. 2C).

### VPA-mediated inhibition of TGF-β1-induced EMT depends on Smad2/3 deactivation

**Fig. 3:**
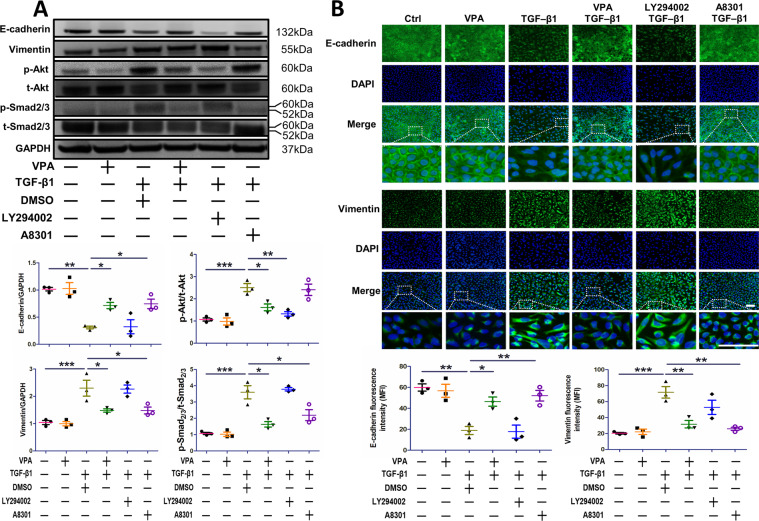
VPA prevents EMT in alveolar epithelial cells induced by TGF-β1 and is dependent on Smad2/3 deactivation. A549 cells were challenged with VPA (1 mM) 72 h prior to TGF-β1. Both LY294002 (20 μM) and A8301 (10 μM) were administered 0.5 h prior to TGF-β1, and cells were subsequently collected 48 h after TGF-β1 stimulation. **A** The expression of E-cadherin, vimentin, p-Akt, and p-Smad2/3 in various conditions. **B** The expression of E-cadherin and vimentin in various conditions was analyzed by immunofluorescent staining. Mean fluorescence intensity was determined by Image J software. Data are presented as dot plot and mean ± SEM (*n* = 3). Scale bar = 50 μm. *P < 0.05; **P < 0.01; ***P < 0.001.

Previous studies suggest that both canonical and noncanonical signaling pathways participate in EMT [34, 35]. We attempted to elucidate the function of both PI3K-Akt and Smad2/3 pathways during EMT in A549 cells. In comparison to TGF-β1-induced EMT, LY294002 was able to reduce the expression of p-Akt (*p* < 0.01) but did not increase E-cadherin or decrease vimentin expression (Fig. 3A, B). On the other hand, A8301 downregulated the expression of p-Smad2/3 compared to the TGF-β1 cohort (*p* < 0.05) and was also capable of inhibiting EMT, as demonstrated by an increase in E-cadherin (*p* < 0.05) and decrease in vimentin expression (*p* < 0.05) in cells incubated with TGF-β1 (Fig. 3A, B).

### VPA and A8301 improve survival, reduce body weight loss, and alleviate lung fibrosis induced by bleomycin

**Fig. 4:**
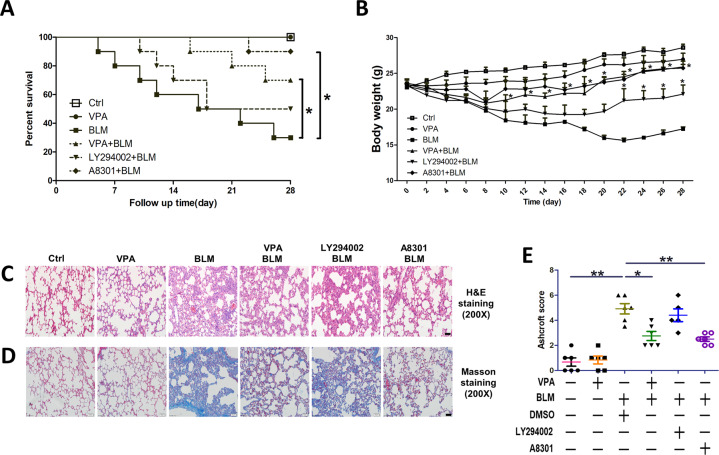
VPA and A8301 improve survival, reduce body weight loss, and mitigate lung fibrosis in mice. Lung fibrosis was induced by endotracheal instillation of bleomycin (2 mg/kg). VPA (100 mg/kg) was administered intraperitoneally daily for 7 days before bleomycin instillation, followed by intraperitoneal administration daily for 28 days. LY294002 (5 mg/kg) or A8301 (1 mg/kg) were administered intraperitoneally once prior to bleomycin instillation. **A** Survival was analyzed by Kaplan–Meier test. *P < 0.05. **B** Change in body weight during the follow-up period. Data are presented as mean ± SEM. *n* = 10. *P < 0.05 compared with bleomycin-induced lung fibrosis. **C** H&E staining of lung slices. **D** Masson staining of lung slices. **E** Ashcroft score of lung tissue. Data are presented as dot plot and mean ± SEM (*n* = 5–6). Scale bar = 50 μm. *P < 0.05; **P < 0.01.

To determine whether VPA is able to protect against lung fibrosis induced by bleomycin, mice were treated with VPA (100 mg/kg) and its effects on survival and body weight was assessed. The survival rate of mice with lung fibrosis was 30% during the 28-day follow-up. However, administration of VPA at 100 mg/kg improved the survival rate in mice instilled with bleomycin (*p* < 0.05) (Fig. 4A). We treated mice with LY294002 and A8301 to assess their effects on survival and body weight in mice, and subsequently compared these results to mice that received VPA. In mice with lung fibrosis, A8301 was shown to increase mice survival during the follow-up period (*p* < 0.05), but LY294002 failed to improve mice survival (Fig. 4A). Similar results were found with body weight, as both VPA and A8301 reduced body weight loss in mice with lung fibrosis (*p* < 0.05). Interestingly, LY294002 did not demonstrate any positive effect on body weight in mice until 22 days after bleomycin instillation (*p* < 0.05) (Fig. 4B).

To further investigate the protective role of VPA against lung fibrosis, histopathological changes in lung tissues were examined. H&E staining (Fig. 4C) and Masson staining (Fig. 4D) demonstrated that VPA was able to reduce structural damage to murine lung tissue. A similar effect was seen with A8301 administration, as Ashcroft scores were decreased compared to lung fibrosis mice (*p* < 0.01), but not LY294002 (Fig. 4E). Collectively, these data suggest that VPA and A8301 protect mice against lung fibrosis induced by bleomycin.

### VPA prevents EMT in fibrotic lung tissue

**Fig. 5:**
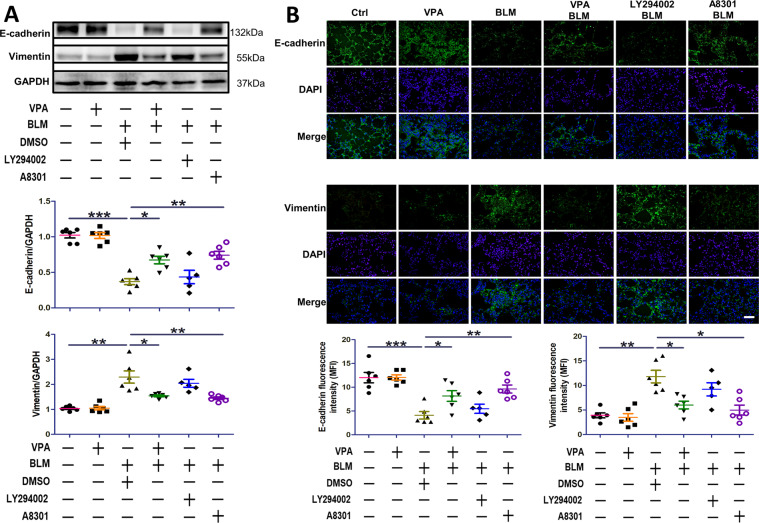
VPA prevents EMT induced by bleomycin in fibrotic lung tissue. **A** The expression of E-cadherin and vimentin in lung tissue from mice. **B** The expression of E-cadherin and vimentin in lung slices were analyzed by Immunofluorescent staining. Mean fluorescence intensity was determined by Image J software. Data are presented as dot plot and mean ± SEM (*n* = 5–6). Scale bar = 20 μm. *P < 0.05; **P < 0.01; ***P < 0.001.

In order to determine whether the protective effects of VPA are related to the prevention of EMT development in mice with lung fibrosis, we assessed the expression of epithelial biomarkers by western blotting and immunofluorescent staining. As demonstrated in Fig. 5A, B, bleomycin induced the downregulation of E-cadherin and upregulation of vimentin compared to mice in the control group (*p* < 0.01). Both VPA and A8301 improved E-cadherin and reduced vimentin expression in fibrotic lung tissue compared to mice with lung fibrosis (*p* < 0.05). These changes were not observed in mice with lung fibrosis that received LY294002.

## Discussion

Our data demonstrate that pretreatment with VPA increases E-cadherin and reduces vimentin expression, indicating the suppression of EMT development in cultured human lung epithelial cells. Furthermore, the administration of VPA downregulates the expression of both p-Akt and p-Smad2/3, while A8301 prevents EMT development. LY294002 fails to demonstrate a similar outcome. The ability of VPA to inhibit EMT was further investigated in a mice model of bleomycin-induced lung fibrosis. VPA administration improves survival and minimizes body weight loss in mice with lung fibrosis. It also leads to a reversal in histopathological changes within the fibrotic lung. Furthermore, VPA upregulates the expression of epithelial biomarkers and downregulates the expression of mesenchymal biomarkers in the fibrotic lung tissue. A8301 treatment demonstrates similar results in this murine model of lung fibrosis.

VPA is widely used as a mood stabilizer to treat various neurological and psychiatric disorders [36, 37]. A previous study demonstrated that VPA inhibits the activity of histone deacetylases in A549 cells following a TGF-β1 challenge [38]. The study also indicated that VPA partially reverses EMT, as suggested by a downregulation in collagen-I expression in comparison to cells incubated with TGF-β1 alone [38]. Our in vitro data illustrate that VPA inhibits EMT in alveolar epithelial cells in a time- and dose-dependent manner. VPA (1 mM) only inhibited EMT when it was administered 72 h prior to TGF-β1 in A549 cells. Interestingly, Kasotakis et al. [39] suggested that VPA has a narrow therapeutic window in the treatment of murine acute lung injury, having demonstrated that VPA must be administered within 3 h to confer a significant improvement in survival in mice. On the other hand, Kabel et al. demonstrated that in rat lung fibrosis, VPA significantly alleviated lung fibrosis when administered continuously for 7 days prior to bleomycin challenge via the downregulation of proinflammatory cytokines and a reduction in nuclear factor kappa-B expression [27]. The data from our in vivo study is consistent with this. Furthermore, we also demonstrate that VPA inhibits EMT within lung tissues. These results suggest that time is a key factor to be considered in order to determine the effects of VPA.

Several signaling pathways are reported to be associated with the development of EMT and pulmonary fibrosis [40–43]. As a component of the canonical pathway, Smad2/3 is activated upon stimulation and subsequently translocates to nucleus, resulting in EMT-related gene transcription, involving transcription factors such as Snail, Twist, and ZEB1/2 [15]. PI3K-Akt represents the noncanonical pathway, which is also activated, and leads to the upregulation of p-Akt during fibrosis [44, 45]. Similarly, our study demonstrates that both p-Smad2/3 and p-Akt are upregulated during EMT of alveolar epithelial cells. Our data also indicate that both pathways are inhibited by VPA during EMT induced by TGF-β1. We further evaluated the functions of Smad2/3 and Akt during EMT and fibrosis by applying inhibitors, and our results indicate that each protein may possess a different role. Similar to VPA, TGF-β type I receptor inhibitor A8301 prevented EMT in alveolar epithelial cells and mitigated lung fibrosis in mice. However, LY294002 did not reverse EMT and failed to protect against pulmonary fibrosis in mice. Whether these key proteins affect each other during lung fibrosis and EMT remains to be elucidated.

Given the fact that complicated signaling pathways are involved in the development of EMT, the interaction and crosstalk between different pathways may contribute to different pathological effects. PI3K-Akt is activated in response to TGF-β1. Therefore, targeting both PI3K and Akt results in the mitigation of TGF-β1-induced EMT and organ fibrosis. Conte et al. reported that LY294002, an inhibitor of PI3K-Akt, inhibits cell proliferation and reduces the expression of α-smooth muscle actin, in addition to collagen production in lung fibroblasts induced by TGF-β1 [46]. Furthermore, in order to avoid its potentially toxic systemic effects, one recent study investigated the efficacy of a PI3K-Akt inhibitor administered via the inhaled route on lung function in fibrosis [47]. The study demonstrated that PI3K-Akt inhibition increased mice survival, reduced collagen accumulation and improved lung function in bleomycin-induced lung fibrosis. Interestingly, it also reduced inflammation and improved lung function in asthmatic mice. Iliopoulos et al. focused on the function of isoforms of Akt rather than the overall activity of Akt during EMT. The results indicate that Akt1 knockdown, but not Akt2, is capable of promoting TGF-β1-induced EMT [48]. Another experiment demonstrated that downregulation of Akt1 contributes to EMT induced by insulin-like growth factor-I or epithelial growth factor, whereas Akt2 downregulation counteracted this effect [49]. Whilst the function of Akt isoforms is important to consider in the context of EMT, the crosstalk between Akt and other signaling pathways makes the situation significantly more complex. Silencing Akt1 promotes EMT and is associated with enhanced extracellular signal-related kinase activation [49]. The direct interaction between Akt and Smad3 prevents Smad3 phosphorylation and further hinders Smad activation induced by TGF-β [50]. Additionally, PI3K has been shown to regulate ubiquitin-mediated proteasomal degradation of Smad2/3. Therefore, the duration of Smad2/3 activation is significantly increased via PI3K inhibition [51]. It may be hypothesized that the PI3K-Akt inhibitor LY294002 failed to protect mice from lung fibrosis and EMT in alveolar epithelial cells due to the differential effects of Akt isoforms, as well as the interaction of Akt on other proteins within the complex signaling network. The precise molecular mechanisms underlying these processes require further investigation.

This study is not without limitations. Firstly, compared to the A549 cell line, the use of primary human lung epithelial cells would have been more appropriate and advantageous for modeling lung fibrosis and to assess the effectiveness of treatment. This should be actively considered in the future. Secondly, the model of pretreatment may limit its clinical use as medication can only be prescribed after diagnosis. However, the theoretical translatability of our findings into clinical practice remains significant due to the fact that cancer patients usually receive chemotherapy postoperatively, hence chemotherapy-induced lung fibrosis can be potentially predicted. Therefore, prophylactic administration of VPA prior to chemotherapy may be a viable option in preventing lung fibrosis and subsequently improving the outcomes of patients with cancer.

In conclusion, our study demonstrated that VPA prevents EMT induced by TGF-β1 in alveolar epithelial cells, which was dependent on Smad2/3 deactivation in vitro. Similarly, pretreatment with VPA attenuates the development of pulmonary fibrosis *via* the inhibition of EMT in mice. This study provides further evidence of the protective effects of VPA and its potential therapeutic value in preventing and treating human lung fibrosis caused by chemotherapy, although these findings require clinical validation in human subjects.

## Data Availability

The datasets analyzed during the current study are available from the corresponding author on reasonable request after publication.
